# Biochemical and Immunological Insights into Capsular Polysaccharide of *Streptococcus pneumoniae* Serotype 38 (American Type 71)—An Emerging Nonvaccine Serotype

**DOI:** 10.1021/acsomega.5c04889

**Published:** 2025-08-18

**Authors:** M. V. N. Janardhan Reddy, Yogeshwar Devarakonda, Rajendar Burki, Kirtimaan Syal

**Affiliations:** † Department of Biological Sciences, Birla Institute of Technology and Science, Pilani, Hyderabad Campus, Jawahar Nagar, Kapra Mandal, Medchal District, Telangana 500078, India; ‡ Research & Development, Biological E Limited, Shameerpet, Hyderabad 500078, India

## Abstract

Invasive pneumococcal disease presents a threat to humankind, predominantly affecting children and the elderly. Despite the availability of high-valency pneumococcal polysaccharide vaccine of PPSV23 (PNEUMOVAX 23) and conjugate vaccines such as VAXNEUVANCE and PREVNAR 20, nonvaccine serotypes continue to contribute to higher mortality rates. The characterization of nonvaccine serotypes is becoming increasingly crucial considering an increase in their prevalence. In this study, biochemical characteristics, immunological properties, and critical quality attributes of the capsular polysaccharide isolated from prevalent nonvaccine serotype 38 (American type 71) have been examined. Advanced analytical techniques, including multiangle light scattering (MALS), ion chromatography, and dynamic light scattering in addition to conventional biochemical methods and SLOTBLOT analysis, were employed. We observed that serotype 38 capsular polysaccharide has a molar mass of 768 kDa with a distribution of 1.5 (±4.5%) and a *z*-average radius of gyration (*R*_g_) of 90 nm. The polysaccharide composition included galactose, *N*-acetylglucosamine, and galacturonic acid, with a distinct peak indicating the presence of the amino acid serine. The near-zero zeta potential measurements indicated that the polysaccharide is either zwitterionic or uncharged. Serotype 38 polysaccharide exhibited immunological cross-reactivity with serotype 5 and serotype 1 polyclonal sera, likely due to a shared epitope region containing a keto sugar component (Sugp) in their repeating units, along with net charge properties similar to serotype 1. These findings revealed novel characteristics of the serotype 38 polysaccharide, including its amino acid composition and net charge, which may contribute to the development of new therapeutics and effective vaccines.

## Graphical abstract

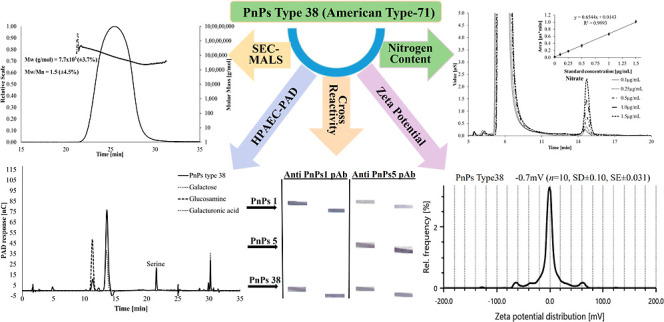

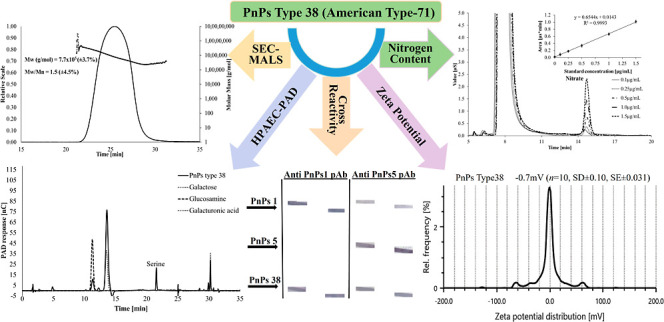

## 1 Introduction

*Streptococcus pneumoniae*, a Gram-positive bacterium with a polysaccharide capsule, can potentially cause both noninvasive and invasive pneumococcal disease (IPD) involving respiratory system predominantly in children under five and the elderly.^1–3^ Though more than 100 serotypes of *S. pneumoniae* are known,^4^ a relatively small subset (less than 30) is responsible for the majority of pneumococcal infections in humans.^1,5^ The diversity of pneumococcal serotypes is primarily determined by variations in the chemical structure of the bacterial capsule’s polysaccharides such as differences in the oligosaccharide units or attached side groups.^5^ The capsular polysaccharide acts as a virulence factor for *S. pneumoniae*, aiding its survival inside the host. Vaccine-mediated immune response against capsular polysaccharides involves protective antibodies with opsonophagocytic activity (OPA).^6–8^ Such antibodies facilitate the complement-mediated uptake and killing of pneumococci by human phagocytic cells.^5,9–12^ Evidently, the introduction of the pneumococcal capsular polysaccharide conjugate vaccine (PCV7) has reduced IPD-related deaths among children under five in the United States from 2000 to 2007.^13^ The PPSV 23 (PNEUMOVAX 23) is a widely used pneumococcal polysaccharide-based vaccine for adults (50 years old and above), whereas the PCV13 conjugate vaccine is preferred for children. The PCV13 was further improvised to PCV20 (PREVNAR 20), which could protect against seven additional serotypes including 8, 10A, 11A, 12F, 15B, 22F, and 33F.^14,15^ The new serotypes that have not been characterized continue to pose challenges due to their unknown capsular polysaccharide structures.^5,16^ The characterization of such new serotypes of *S. pneumoniae* involving examination of the biological and serological characteristics of capsular polysaccharides may facilitate their inclusion in the vaccine designs. The coverage of existing PCV 10, 13, 20 and PPSV 23 vaccines has been observed at 16%, 24%, 48%, and 66%, respectively.^17^ Moreover, approximately 30% of the total *S. pneumoniae* isolates belong to nonvaccine serotypes (NVTs), highlighting the need to include these serotypes in vaccine coverage to improve population protection.^17,18^ The prevalence of pneumonia caused by nonvaccine serotypes varies globally.^4,18,19^ In 2019 alone, an estimated one million deaths among children under five were attributed to pneumonia.^20^ In India, around 21% of the IPD cases in 2016 were attributed to nonvaccine serotypes.^18^

The nonvaccine *S. pneumoniae* serotypes are continuously emerging as evident by their increased incidence rates and spread.^19^ The most common serotypes that infect children include 10A/F, 7C, 35A/B, 16F, 19A, 3, and 38.^19,21^ Surveillance data from countries such as the USA, Australia, Finland, France, Norway, Canada, and Ethiopia indicate that serotype 38 has become more prevalent causing IPD across all age groups.^19,21–23^ In India, Manoharan et al. identified serotype 38 in more than 1% of the *S. pneumoniae* clinical isolates.^24^ The genetic analysis of the capsular biosynthetic locus of *S. pneumoniae* serotype 38 has been characterized and described elsewhere.^25^ Surveillance data (year 2025) showed the increased IPD cases of serotype 38 in Germany and Poland.^26^ Together, the understanding of its CPS biochemical characteristics, immunological properties, and critical quality attributes (CQAs) has become vital, considering its widespread distribution, high incident rates, and identification in clinical isolates.

Recently, Li et al. determined the repeating sequence of the type 38 polysaccharide by NMR analysis as →3)-[β-D-Galf(1 → 2)]-β-D-GalpA6­(l-Ser)-(1 → 3)-α-D-GlcpNAc-(1 → 3)-α-D-Sugp-(1 → 4)-α-D-Galp­(2OAc)-(1 →^27^. In this study, alongside biochemical characterization, the net charge was determined, nitrogen-based process impurities and phosphorus content were estimated, proposed critical quality attributes (CQAs) were identified, and immunological cross-reactivity with selected serotypes was assessed—collectively offering key insights for developing effective therapeutic strategies against IPD caused by serotype 38.

## 2 Experimental Process

### 2.1 Materials

Purified pneumococcal polysaccharide serotype 38 was procured from the American Type Culture Collection (ATCC 543-X). Glucose (Glc), galactose (Gal), mannose (Man), fucose (Fuc), rhamnose (Rha), glucuronic acid (GlcA), galacturonic acid (GalA), glucosamine (GlcN), galactosamine (GalN), *N*-Acetylglucosamine (GlcNAc), *N*-acetyl galactosamine (GalNAc) and BSA monomer were purchased from Sigma-Aldrich Co. (St. Louis, USA). *N*-Acetyl fucosamine (FucNAc) and *N*-acetyl-pneumosamine (PneNAc) were purchased from Omicron Biochemicals Inc. USA. Trifluoroacetic acid (TFA) and hydrofluoric acid (HF) were obtained from Merck, India. Sodium acetate and sodium hydroxide 50% solution were purchased from Sigma-Aldrich (St. Louis, USA). Mouse monoclonal antibodies of serotypes 1 and 5 were procured from AbMax-China, and rabbit polyclonal antisera and capsular polysaccharide of serotypes 1 and 5 were procured from Statens Serum Institute Diagnostica, Denmark. Secondary antibodies of goat antimouse IgG peroxidase and antirabbit IgG peroxidase purchased from Bio-Rad laboratories India. All chemicals were ACS reagent-grade and IC-grade with a purity specification of ≥90–99% (Fluka, Sigma-Aldrich).

### 2.2 Methods

#### 2.2.1 Preparation of CPS of Serotype 38 and the Sugar Standards

The stock solution of purified polysaccharide of serotype 38 was prepared by dissolving it in ASTM Type-II water at a concentration of 2 mg/mL (w/v). All other sugar stock solutions were prepared as 1 mg/mL (w/v) in ASTM Type-II water, which were subsequently diluted to the final concentration of 10 μg/mL (for each sugar). The buffered serotype capsular polysaccharide stock, which originally had sodium chloride in its lyophilized form, was subjected to a desalting procedure wherever needed.

#### 2.2.2 SEC-UV-MALS-RI

The pneumococcal polysaccharide (PnPS) physical parameters such as molar mass were determined by SEC-UV-MALS-RI. It is crucial to maintain the molecular size (MS) of PnPS within the specified range for the design of conjugate vaccines either through physical or acid hydrolysis before using it for conjugation.^28,29^ The purified polysaccharide obtained from ATCC was initially dissolved in ASTM Type-II water to achieve a concentration of 2 mg/mL (w/v). Subsequently, a 100 μL aliquot of this solution was injected into a size exclusion chromatography (SEC) column that was connected to UV-MALS-RI detectors. The scheduled calibration was carried out for multiangle light scattering (MALS) using toluene (batch mode). We have used a BSA monomer in PBS (pH-7) for the calibration of MALS detector coupled with the size exclusion chromatography before sample analysis as per user manual instructions (Waters/Wyatt Technical Note: TN3506 Molecular Standards for Determining System Constants and Validating SEC-MALS-IV and FFF MALS Systems, 2018). BSA standard has consistent molar mass and well-defined refractive index increment (d*n*/d*c*) of ∼0.185 mL/g in PBS (pH 7). Its radius <10 nm enables isotropic light scattering that is evenly distributed across angles, thereby improving the precision of molar mass calculations. The HPLC system utilized for the analysis consisted of SHODEX SB 806HQ and SHODEX SB 803HQ connected in series with 100 mM sodium phosphate and 0.05% sodium azide pH 7.2 serving as the mobile phase. The flow rate of 0.5 mL/min of the mobile phase was maintained through the connected analytical columns. The Agilent 1260 series HPLC, equipped with miniDAWN TREOS and Optilab T-rEX RI detectors from Wyatt Technology, USA, was used to carry out the analysis. The molar mass and size distribution pattern was determined using the refractive index as a concentration source-detector by inputting a d*n*/d*c* of 0.133 mL/g^30,31^ to the ASTRA software.

#### 2.2.3 HPAEC-PAD

10 μg/mL polysaccharide treated with 10 M TFA with the ratio of 4:1 (v/v) in a glass vial, sealed with caps equipped with a Teflon-faced rubber insert, was subjected to incubation at 121 °C for 2 h in a dry bath with a safety lid. Post incubation, the sample was subjected to nitrogen evaporation set at 45 °C for 30 min to evaporate the TFA. The polysaccharide smear in the vial was then dissolved in 1 mL of ASTM Type-II water and then filtered through a 0.22 μm syringe filter into the HPLC vial. In a parallel set, 10 μg/mL polysaccharide was first treated with HF followed by TFA as per the referred protocol.^32^ 20 μL of depolymerized polysaccharide and sugar standards were injected into a Carbopac PA10 (4.6 × 250 mm) column and guard (4.6 × 50 mm) connected to the ICS-5000 (Thermo Fisher Scientific, Sunnyvale, CA, USA) system equipped with an electrochemical detector that includes an Ag/AgCl reference electrode and a disposable gold working electrode. The mobile phase of A; 18 mM NaOH, B; 100 mM NaOH, and C; 1 M sodium acetate in 100 mm NaOH were pumped into the column as a gradient program with the flow rate of 1 mL/min. The temperatures of the column and autosampler (ASAP) were set at 30 and 15 °C, respectively. The carbohydrate (standard quad) waveform was applied to the electrochemical detection (ECD) and the data were processed using Chromeleon software.

#### 2.2.4 HPAEC-CD

The O-acetyl content determines the immunogenic response of the polysaccharides as reported.^33,34^ The acetyl content of the capsular polysaccharides was examined. The polysaccharide at a concentration of 200 μg/mL was treated with 0.1 N NaOH and incubated at 37 °C for 2 and 4 h. Following the incubation, the mixture was passed through 10 kDa nanosep centrifugal filters that had been prerinsed three times with ASTM Type-II water at 10,000 rpm for 15 min. To avoid any dilution of the sample filtrate with droplets from the filter holder used for the prerinse process, the filtrate was collected into a fresh 10 kDa filter holder. A working standard was devised using sodium acetate (as acetate source) in a concentration range of 0.625 to 40 μg/mL. The standard was diluted with ASTM Type-II water, and the sample filtrate was analyzed as described elsewhere.^35^

The total nitrogen (contribution from hexosamines and residual protein impurity) content in the polysaccharide was estimated as per the referred protocol.^36^ The nitrogen content is not solely attributed to hexosamines but is also influenced by any protein content present in the capsular polysaccharides and therefore may help in estimating protein impurities. In addition to amines, phosphates may also exist as sugar derivative components, such as ribitol phosphate and ribose phosphate, within these polysaccharides. Moreover, nucleic acid impurities in the purified capsular polysaccharides may contribute significantly to the overall nitrogen and phosphorus levels. Therefore, it is essential to quantify these components within the polysaccharides in order to provide indirect insights into the impurity levels of residual proteins and nucleic acids, serving as an orthogonal verification method alongside conventional OD 260/280 measurements. In this study, 200 μg/mL serotype 38 polysaccharide was subjected to digestion using 0.5 M potassium persulfate in 0.5 M NaOH at 100 °C for 16 h. Following digestion, the sample was diluted before analysis by HPAEC-CD. The analysis was performed using a Thermo ICS-5000 system, equipped with an IonPac AS15 guard column (50 × 4 mm) and an IonPac AS15 analytical column (250 × 4 mm), to separate and estimate the nitrogen and phosphorus content as previously reported.^36^

#### 2.2.5 Orthogonal Verification of Sugar Composition and Its Derivatives

The bacterial capsular polysaccharide structure comprises common sugar derivatives such as methyl pentoses and uronic acids, along with the common sugar forms of hexoses and pentoses. The sugar composition obtained by HPAEC-PAD was verified by the conventional colorimetric methods such as the Cysteine-HCl method for methyl pentoses^37^ and the Carbazole method for uronic acids.^8,38,39^ As reported, rhamnose was used as a standard for the methyl pentose assay, and glucuronic acid was used as a standard for the uronic acid assay. Briefly, for the methyl pentose assay, 100 μL of rhamnose standard ranging from 2 to 40 μg/mL was prepared alongside the diluted sample. Then, 500 μL of reagent A (1 in 6 v/v Water/H_2_SO_4_) was added to the standard solutions and the diluted sample followed by incubation at 90 °C for 7 min in a water bath. Then, 40 μL of reagent B (3% w/v Cysteine HCl) was added to all tubes and incubated at 37 °C for 30 min. After incubation, 200 μL of each standard and sample were transferred into a 96-well plate, and the absorbance was measured at 396 and 430 nm. As previously reported, the Carbazole method was used for estimating the uronic acid content. Briefly, 100 μL of glucuronic acid standard range from 5 to 50 μg/mL was prepared, and 100 μL of diluted sample was added to 500 μL of reagent A (0.030 M Borate buffer/H_2_SO_4_) and incubated at 90 °C for 15 min in a water bath. After the addition of 20 μL of reagent B (0.125% Carbazole in ethanol), all tubes were incubated at 90 °C for 15 min. After incubation, 200 μL of each standard and sample were transferred to a 96-well plate, and the absorbance was measured at 530 and 660 nm.^38,39^

#### 2.2.6 Dynamic Light Scattering

The zeta potential was measured in series mode using Omega Cuvettes with the light scattering instrument of Litesizer-500 (Anton Paar, India) by Kalliope software, and the default measurement parameters were set.^40^ The lyophilized polysaccharide powder of serotype 38 had high sodium chloride content (6 M), which interfered with the charge measurement. Therefore, the polysaccharide powder was dissolved in ASTM Type-II water and desalted by passing it through a 10 kDa cutoff centrifugal filter to remove the sodium chloride content. Sodium chloride hinders the zeta potential measurement due to its high conductivity and may lead to charring of the electrode in cuvette. The buffer exchanged sample was further diluted to 200 μg/mL with ASTM Type-II water to meet the instrument measurement specifications of the sample such as accepted filter optical density and sufficient mean intensity (>20 kcounts/s) values. Before analyzing the sample, the instrument was calibrated with the known zeta potential standards.

#### 2.2.7 SLOTBLOT

The capsular polysaccharide of serotype 38 consisted of a keto sugar in its repeating unit identified as “Sug_*p*_” as described in ref 27. Evidently, a keto sugar (2-acetamido-2,6-dideoxy-d-xylo-hexos-4-ulose) was also present in another pneumococcal serotype i.e. PnPS serotype 5.^41^ Additionally, PnPS serotype 1 also featured a unique and rare amino sugar moiety, AAT sugar (2-acetamido-4-amino-2,4,6-trideoxy-d-galactose), which was previously noted for its immunogenicity.^42,43^ Therefore, we tested whether the serotype 38 polysaccharide may elicit antibodies functionally similar to those elicited by the polysaccharides derived from serotypes 1 and 5. Such antibody response is expected only if the keto sugar (sug_p_) acted as an antigenic epitope in the serotype 38 polysaccharide. We performed a SLOTBLOT assay to test our hypothesis^44^ with the aim to evaluate the cross-reactivity of serotype 38 against antibodies specific to serotypes 1 and 5. Briefly, PnPS serotypes 1, 5, and 38 were adhered to a nitrocellulose membrane at two different concentrations in four sets. The 1% skim milk powder solution in PBS was used as a blocking reagent over the membrane. Following blocking, the membrane was washed three times with PBST (PBS with 0.05% Tween 20). Optimized dilutions of monoclonal and polyclonal antibodies specific to serotypes 1 and 5 were added to designated blots and incubated for 1 h. After incubation, the membrane was washed again with PBST and incubated with the respective secondary antibodies labeled with horseradish peroxidase. The reaction was developed using a TMB substrate to visualize antibody binding on the membrane concerning the lanes of the loaded antigens.

#### 2.2.8 Critical Quality Attributes of Capsular Polysaccharides

Capsular polysaccharides content should meet stringent standards for identity, purity, potency, and safety set by regulatory authorities like the FDA, EMA, and WHO for inclusion in vaccine design.^45^ The vaccine’s reproducibility depends on various factors including surface charge that affects stability and particle distribution. Based on the regulatory guidelines, monitoring and adhering to critical quality attributes (CQAs) are essential for characterization of antigenic content and demonstration of their immunogenic properties in order to ensure optimal efficacy and protection against the corresponding pneumococcal serotype. The chemical composition, including sugars and functional groups, must be precise to maintain the immunogenic properties and mimic the native bacterial surface. Establishing specific percentage limits for each functional group within the polysaccharides is necessary to evaluate the integrity of the immunogenic functional groups and confirm the preservation of the vaccine’s immunogenic characteristics. The CQAs for these polysaccharides include several factors that can significantly impact the performance and reliability of the final vaccine product. The most important attributes to assess the PnPS quality are molecular size, purity, sugar composition, and surface charge. The polysaccharides that are too small may be ineffective, while those too large can complicate manufacturing due to excessive viscosity. Consistent molar mass and size distribution are also necessary for uniform vaccine quality.^28,29^ Impurities such as proteins, nucleic acids, and endotoxins may reduce the vaccine effectiveness and safety. Rigorous purification is essential to minimizing these contaminants. Ensuring that these CQAs are met is crucial for producing safe and effective vaccines. Hence, we attempted to propose the limits of CQAs with the ATCC-purified polysaccharide, as it is used as a reference standard by the manufacturers to develop immunological-related methods.

## 3 Results and Discussion

### 3.1 Size and Molar Mass by SEC-MALS

The molar mass and size distribution patterns were determined by SEC-MALS analysis. The purified Spn type 38 CPS (2 mg/mL) was injected into size exclusion columns connected to an Agilent HPLC system equipped with a UV-MALS-RI detector. The normalization was carried out with the BSA monomer prior to analysis. The refractive index increment, d*n*/d*c*, of the polysaccharide (0.133 mL/g), was used to determine the molar mass, rms radius moments (rz), and size distribution pattern by using ASTRA software. The polysaccharide peak was observed between 22 and 30 min, with the size distribution graph showing a range of 1.0 × 10^7^ to 1.0 × 10^5^ g/mol (Figure 1). The average molar mass of the purified CPS of Spn type 38 was determined to be 7.7 × 10^5^ g/mol (±3.7%) with a polydispersity (*M*_w_/*M*_n_) value of 1.5 (±4.5%) and *z*-average radius of gyration (Rz) value of 90 nm. These results can be used as a reference value for assessing the consistency of the purification process, in terms of both fermentation and downstream purification, for purified CPS of Spn type 38.

**Figure 1:**
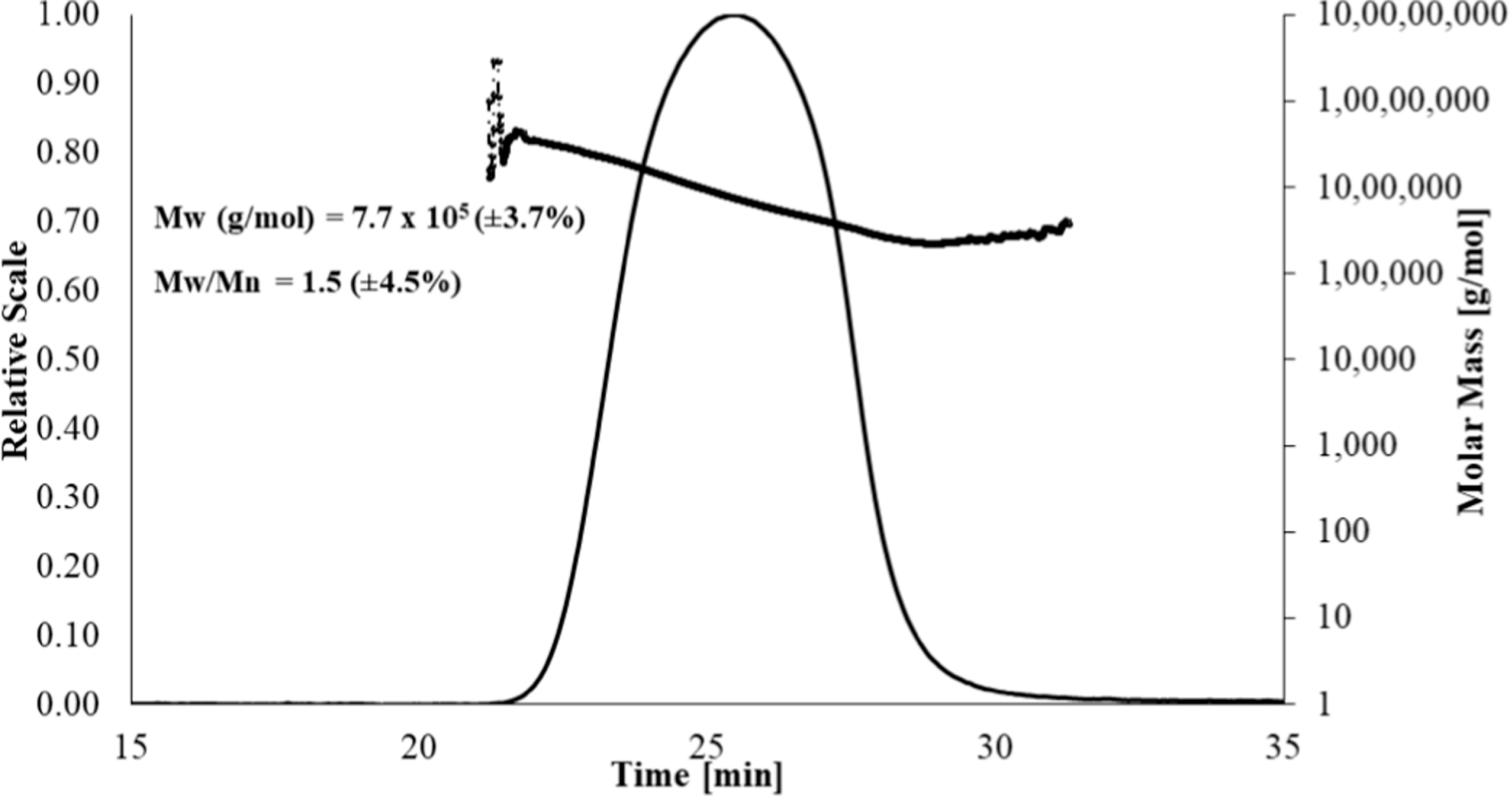
SEC-MALS profile of molar mass vs time of PnPS serotype 38.

### 3.2 Monosaccharide Composition by HPAEC-PAD

As described in the Methods section, the depolymerized samples were analyzed in an ion chromatography system using a CarboPac PA10 column coupled with electrochemical detection (ECD). The monosaccharide components identified in the CPS included *N*-acetylglucosamine, galactose, and galacturonic acid, alongside an unidentified peak with a retention time of 22 min (Figure 2A,B). With the aim to elucidate the nature of the unidentified peak (RT of 22 min), individual injections of various monosaccharides, including fucose, glucose, galactose, mannose, glucosamine, fucosamine, pneumosamine, and galacturonic acid, were performed (Figure 2A). Based on the retention time of the unidentified peak, which eluted at approximately 50 mM sodium acetate in 100 mM NaOH, it was inferred that this peak was not attributable to any monosaccharide or disaccharide, as all such compounds elute from the CarboPac PA10 column with 100 mM sodium hydroxide alone, without the necessity of sodium acetate.^46^ The unidentified peak’s elution in the presence of sodium acetate suggested it might be a sugar acid or a nonsugar compound. It is hypothesized that this peak represents a sugar acid or a compound containing an amino acid, potentially forming a peptide bond with galacturonic acid or another amine present in the CPS repeating unit. As we were in the process of characterizing this unidentified compound, Li et al. reported^27^ structural studies of the serotype 38 capsular polysaccharide and they identified the unknown component as the amino acid serine conjugated to galacturonic acid. We also confirmed the presence of serine. The discovery represents the first instance of an amino acid being identified within the repeating unit of a *S. pneumoniae* capsular polysaccharide. Together, we have identified monosaccharide peaks in the chromatogram as galactose, *N*-acetylglucosamine, and galacturonic acid, in addition to the amino acid-serine. The elution pattern and the retention time of each component have been summarized in Table 1. Additionally, a minor set of peaks was observed between 5 and 8 min (Figure 2C), which overlaid with the peaks (other than the main known sugars) obtained for PnPS serotype 5 hydrolyzed with HF followed by TFA, for serotype 38 (Figure 2C zoomed chromatogram). This overlaying confirmed and supported the recently reported work^27^ that these peaks belong to the Sug_*p*_ (2-acetamido-2,6-dideoxy-d-xylo-hexos-4-ulose) moiety present in PnPS serotype 38. This novel structural feature may influence host–pathogen interactions and immunogenicity or present challenges in conjugation chemistry used for coupling with carrier proteins. Further investigation is warranted to explore these implications comprehensively.

**Figure 2:**
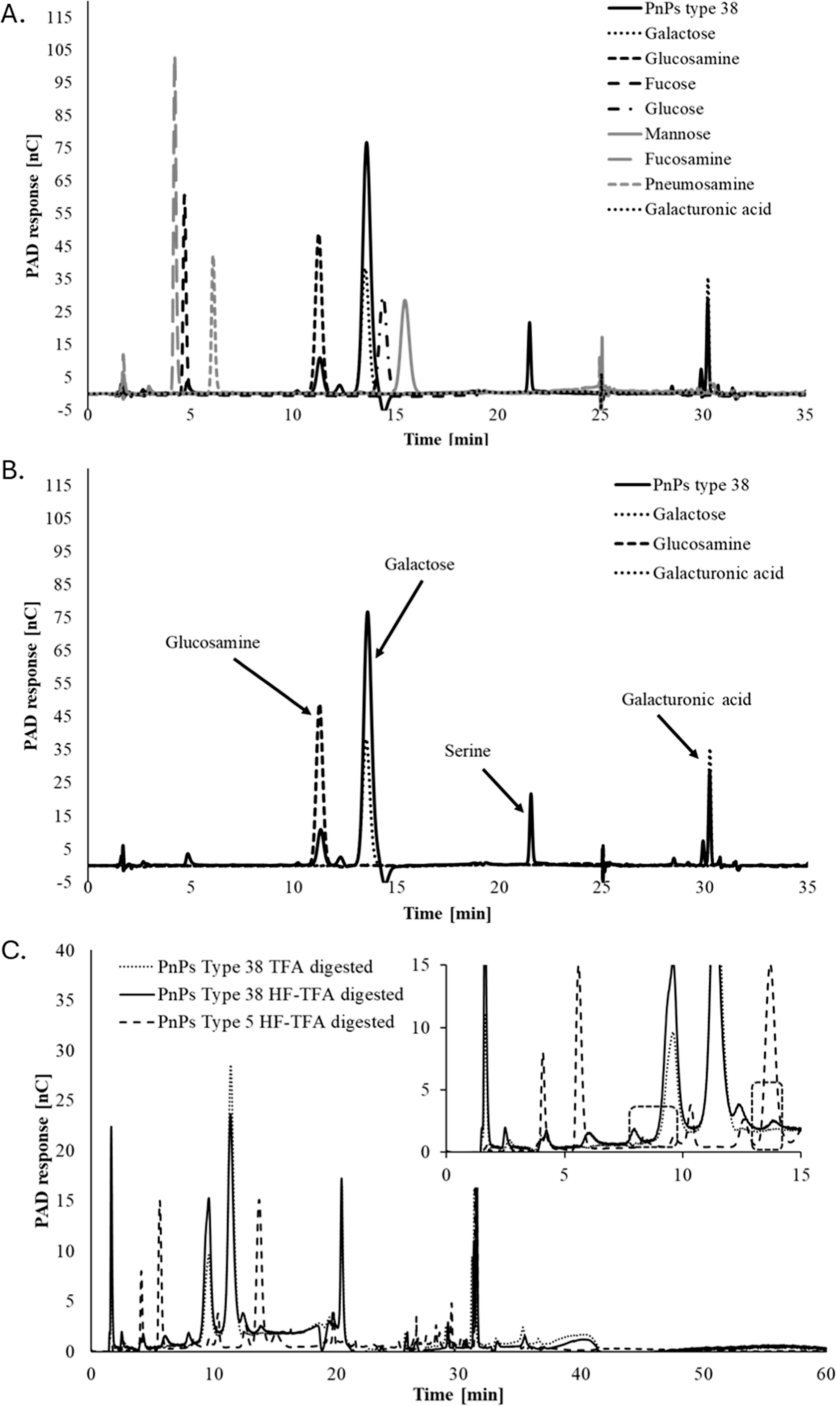
(A) Overlaid chromatogram of TFA hydrolyzed PnPS type 38 and the individual monosaccharides injected into the CarboPac PA10 column connected to HPAEC-PAD. (B) Overlaid chromatogram of TFA hydrolyzed PnPS type 38 and its respective monosaccharides injected into the CarboPac PA10 column connected to HPAEC-PAD. Serine peak was also confirmed by the application of serine standard. (C) Overlaid chromatogram of TFA digested PnPS type 38, HF followed by TFA hydrolyzed PnPS type 38 and PnPS type 5 injected into the CarboPac PA10 column connected to HPAEC-PAD along with zoomed profile from 0 to 15 min (Inset).

**Table 1:** Retention Times of Key Components Identified during Chromatographic Analysis^a^

Peak number (*V*_0_ to *V*_t_)	Component name	Retention time [minutes]
1	glucosamine	11.3
2	galactose	13.6
3	serine	21.6
4	galacturonic acid	30.2

a Each peak corresponds to a specific compound eluted between the void volume (*V*_0_) and total volume (*V*_t_). Retention time is measured in minutes and reflects the time each component takes to pass through the chromatographic column under the specified conditions.

### 3.3 O-Acetyl Content by HPAEC-CD

The O-acetyl content is one of the important immunogenic functional groups for capsular polysaccharide-based vaccines.^33,34^ Hence, it is mandated to quantify it as the quality attributes of the CPS. In the ATCC-purified polysaccharide of serotype 38, under mild alkaline conditions, as mentioned previously, the O-acetyl groups were selectively hydrolyzed and released as acetate ions. The hydrolysate acetate was separated from other ions by the Thermo ICS-5000 system equipped with a strong anion exchange column, and a guard column of IonPac AS11-HC guard column i.d. (50 × 4 mm) and an IonPac AS11-HC analytical column i.d. (250 × 4 mm) were used and estimated the content. The equimolar ratio of sodium acetate as acetate was used as an assay standard for the range 0.625–40 μg/mL (Figure 3A). The polysaccharide was kept under mild alkaline conditions, 10 mM NaOH at 37 °C, for 2 and 4 h to confirm the complete release of O-acetyl groups from the polysaccharide. The standard acetate peak has a retention time of 7.2 min, which was overlaid with the acetate peak from the sample. The peak area obtained for two and four hours of incubation was observed to overlay (Figure 3B) and the O-acetyl content was calculated to be same for both two hours and four hours of incubation with a value of 5.8%. The O-acetyl content reported by other techniques of HPAEC-CD in our paper represents the overall percentage of the O-acetyl content against total polysaccharide mass (w/w) and not the percentage of O-acetylated repeating units in a total polysaccharide chain as described by Li et al.^27^ This methodological and interpretive distinction explains the observed differences in the reported values.

**Figure 3:**
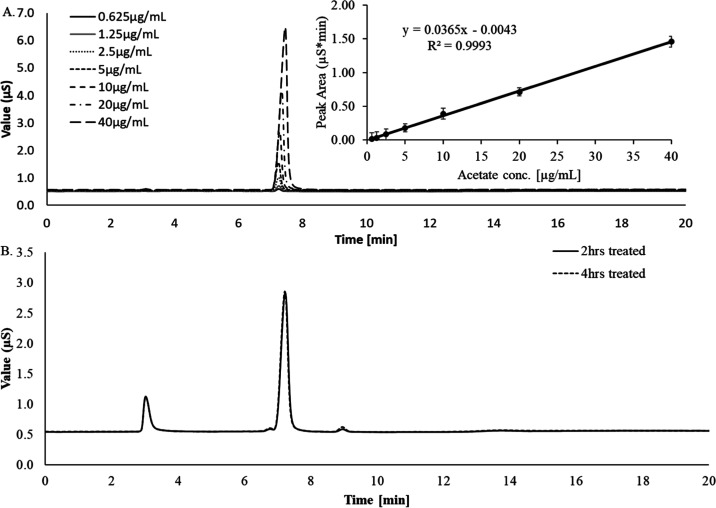
(A) Overlaid profiles of acetate standards (0.625 to 40 μg/mL) from HPAEC-CD and are separated by using an AS11 HC column and 5 mM NaOH as an eluent. (B) Overlaid profiles of PnPS type 38 from HPAEC-CD analysis of acetyl groups released for two and four hours of mild alkaline treatment and are separated using an AS11 HC column and a 5 mM NaOH eluent.

### 3.4 Polysaccharide Net Charge by DLS

As described in the Methods section, the sample was diluted to 200 μg/mL in ASTM Type-II water to meet instrument specifications, including acceptable optical density and mean intensity (>20 kcounts/s). With default settings, zeta potential measurements were carried out in series mode of ten readings with the Litesizer-500 (Anton Paar, India) using Omega Cuvettes and Kalliope software. The zeta potential of the polysaccharide of serotype 38 was found to be −0.7 mV (Figure 4). The PnPS type 1 is categorized and reported as a zwitterionic polysaccharide elsewhere.^47,48^ With the aim to compare, we measured the zeta potential for the PnPS type 1 at 200 μg/mL in ASTM Type-II water and it also showed a value of −0.9 mV. This value also falls within the −10 to +10 mV range, potentially indicating a neutral or zwitterionic nature. We also studied the zeta potential of nonzwitterionic polysaccharide of serotype 5 and obtained the value of −46.5 mV (data not shown). The absolute zeta potential values exceeding ±30 mV are classified as strongly cationic or anionic.^49^ PnPS serotype 1 was classified as a zwitterionic polysaccharide, and the similar charge of PnPS serotype 38 could imply a zwitterionic or net neutral charge. This information is crucial for understanding the type 38 polysaccharide’s behavior in vivo and stability in different formulations. The near-neutral surface charge may influence its interactions with the antigen-presenting cells (APCs) and ZPS-mediated T cell activation and may trigger the T cell-dependent B cell immune response.^47^

**Figure 4:**
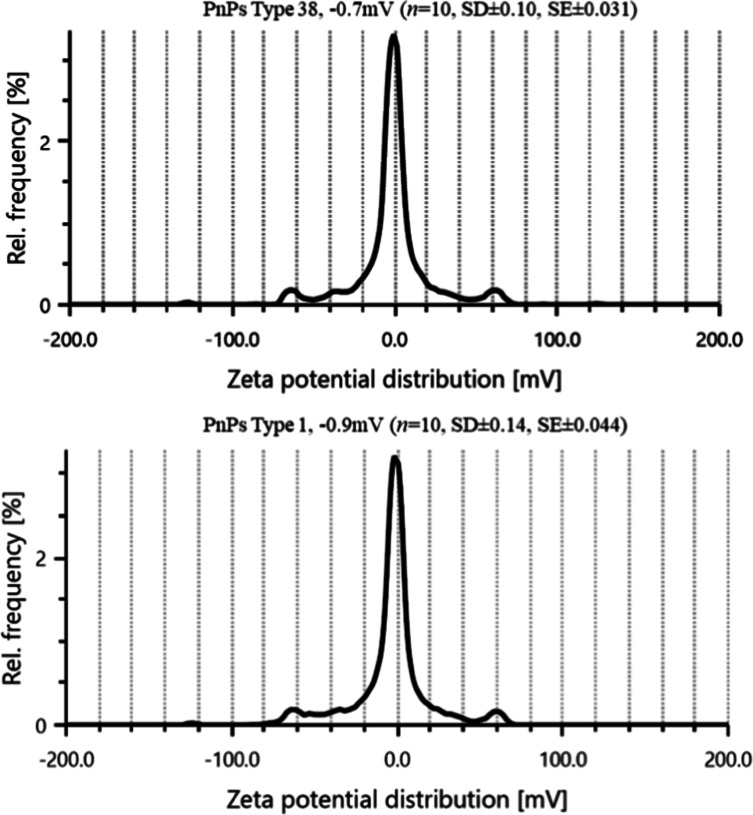
Zeta potential distribution profile for PnPS serotype 38 and serotype 1. Both serotypes exhibited similar net charge values with their zeta potentials centered near 0 mV. The near-identical charge value of PnPS serotype 38 also suggested it might be in a zwitterionic state or net neutral polysaccharide.

### 3.5 Orthogonal Verification of Sugar Derivatives by Conventional Methods

The sugar composition obtained by HPAEC-PAD for PnPS serotype 38 was orthogonally verified further with the conventional biochemical methods for methyl sugars (Rhamnose) and sugar acids (Uronic acid) by following the procedures reported and described previously. A good linear curve was observed with a regression value of >0.99 for both methods (Figure 5A,C). The OD values observed in the PnPS serotype 38 sample tested at three different concentration levels of 100, 200, and 400 μg/mL were comparable to blank, thereby confirming the absence of methyl pentoses in serotype 38 capsular polysaccharide (Figure 5B). It was supported by the monosaccharide profiles obtained by HPAEC-PAD as there was no rhamnose peak in the chromatogram. For uronic acid content evaluation, the sample was tested at three different concentrations, and the obtained average uronic acid content was determined to be 7.7 (Figure 5D).

**Figure 5:**
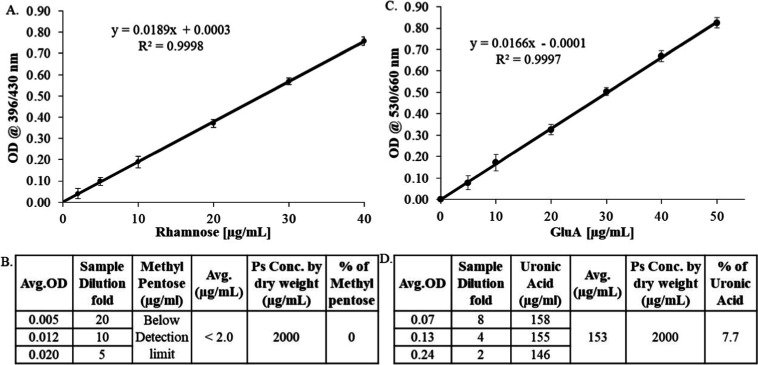
(A) Linear standard curve for rhamnose obtained using the Cysteine-HCl method. (B) Sample results showing the percentage of methyl pentoses tested at three different concentration levels. (C) Linear curve for uronic acid determined using the Carbazole method. (D) Results table displaying the percentage of uronic acid content in the sample, tested at three different concentration levels.

### 3.6 Nitrogen and Phosphorus Content by HPAEC-CD

Amines (hexosamines) and phosphorus-containing sugars (ribose-5-phosphate and ribitol-5-phosphate) are important attributes of capsular polysaccharides.^5^ In addition to amines, protein impurities in the polysaccharides also contribute to the overall nitrogen content. Similarly, nucleic acid impurities add to the total phosphorus content. With the aim to determine the nitrogen and phosphorus content in the capsular polysaccharide sample, PnPS serotype 38 was subjected to persulfate hydrolysis followed by HPAEC-CD. The quantification was performed against standard linear calibration curves established with urea for nitrogen content and sodium phosphate monobasic for phosphorus content (Figure 6A,B). In the chromatogram, a nitrate peak was detected, whereas no phosphate peak was observed in the sample (Figure 6C). The nitrogen content was estimated to be 3.67%, with no detectable phosphorus content. Expectedly, the phosphorus content was undetectable, confirming the minimal nucleic acid contamination in the tested capsular polysaccharide (CPS).

**Figure 6:**
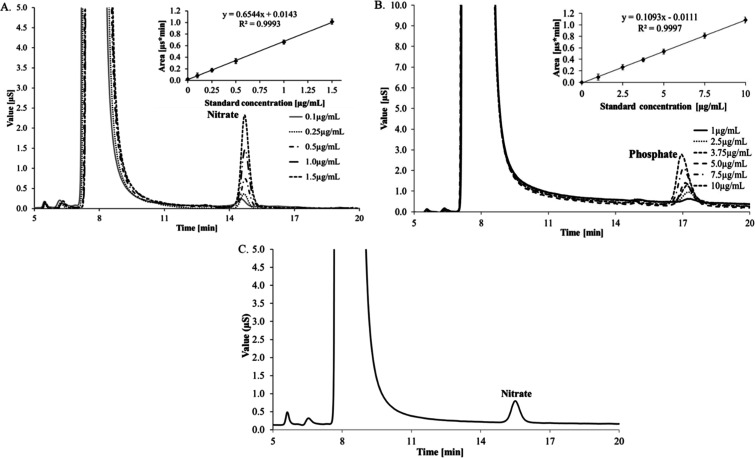
(A) Overlaid chromatographs of standard concentrations and linearity curve of nitrate (0.1 to 1.5 μg/mL). (B) Overlaid standards and linearity curve of phosphate (1.0 to 10 μg/mL). (C) Chromatogram of PnPS type 38. All standards and samples were injected into the Ion PAC AS15 (4 × 250 mm) column with guard (4 × 50 mm) by pumping the 38 mM sodium hydroxide as a mobile phase with the flow rate of 1.2 mL/min and detected with suppressed conductivity detection, ADRS auto suppression recycle mode, 113 mA current.

### 3.7 Immunochemical Properties

With an aim to assess the validity of our hypothesis that the keto sugar (Sug_p_) moiety of serotype 38 polysaccharide may constitute a portion of an epitope recognized by the host immune system, we compared the HPAEC-PAD profiles and sugar composition of PnPS serotype 38 with those of serotype 5. Overlapping unidentified peaks were observed, potentially generated from the sug_p_ by acid hydrolysis using hydrofluoric acid (HF) followed by trifluoroacetic acid (TFA) (Figure 2C). As described earlier, the CPS of Spn type 38 has a net charge of −0.7 mV, indicating that it is in a zwitterionic form like a CPS of Spn type 1,^47,48,50^ which has a net charge of −0.9 mV (Figure 4). Therefore, we performed the SLOTBLOT assay using serotype 1 and serotype 5 monoclonal antibodies from AbMax and polyclonal antibodies from SSI, both of which possess rare sugars and a zwitterionic form of their polysaccharide repeating units. Our results revealed a clear blue blot upon the addition of the TMB substrate in the slot where the serotype 5 and serotype 1 polyclonal antibodies were loaded against the serotype 38 lane onto the membrane (Figure 7). In each set, lanes 1, 2, and 3 corresponded to PnPS 1, 5, and 38, respectively, with the left spots (2nd vertical line) representing the 2 μg/mL concentration and the right spots (1st vertical line) representing the 1 μg/mL concentration. The intensity and pattern of the spots in each lane and concentration provide insights into the antibody specificity and binding affinity for the respective PnPS. These findings provide evidence supporting the hypothesis that serotype 38 possesses an epitope characteristic similar to that of serotype 5, and partially similar to that of serotype 1, as the rare sugars (Table 3) in its capsular polysaccharide repeating unit. Consequently, we anticipate that serotype 38 may offer cross-protection against invasive pneumococcal disease caused by serotype 5 and vice versa subjected to further testing.

**Figure 7:**
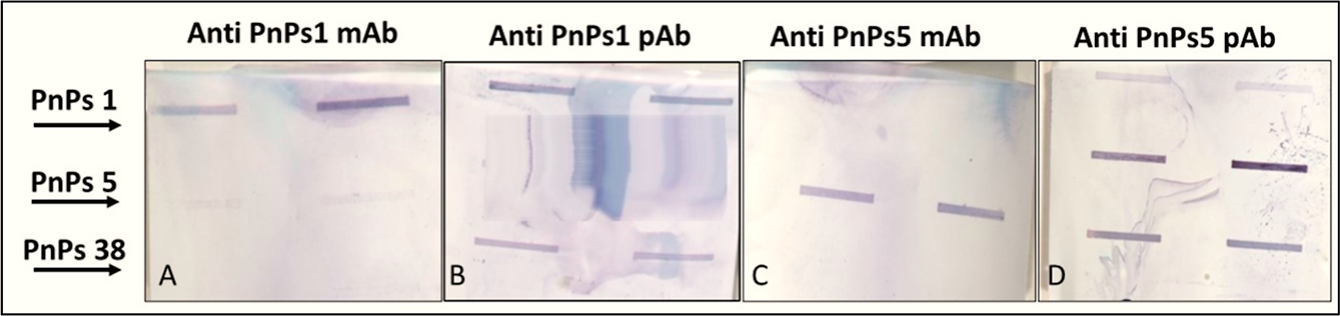
SLOTBLOT analysis-the horizontal lanes 1, 2, and 3 represented PnPS 1, 5, and 38, loaded in, respectively, at two different concentrations: 1 μg/mL and 2 μg/mL. The analyses were performed across four sets (A, B, C, D). The blots of A & B were tested with Anti-PnPS type1 monoclonal antibodies (mAb) and polyclonal antibodies (pAb), respectively. (C,D) were tested with Anti-PnPS type 5 monoclonal antibodies and polyclonal antibodies respectively.

The findings of this study revealed crucial insights into the immunological characteristics of *S. pneumoniae* serotype 38, particularly its interaction with antibodies from other serotypes. Notably, serotype 38 exhibits cross-reactivity with polyclonal antibodies of serotypes 5 and 1, yet it does not react with the corresponding monoclonal antibodies. This discrepancy highlights the complexity of the immune response and the potential structural similarities and differences in capsular polysaccharides among these serotypes.^51^ The cross-reactivity observed with polyclonal antibodies indicates that there are shared epitopes between serotype 38 and serotypes 5 and 1. The polyclonal antibodies, being a heterogeneous mix, could recognize multiple antigenic sites on a pathogen. This broad recognition suggested that certain polysaccharide structures within serotype 38’s capsule were similar enough to those in serotypes 5 and 1 to elicit an immune response. Such shared epitopes might include common sugar components or structural motifs that were conserved across these serotypes.

In contrast, the lack of reaction with monoclonal antibodies, which were specific to a single epitope, suggested that the unique epitopes targeted by these antibodies in serotypes 5 and 1 were absent or significantly different in serotype 38. This implies that while there are similarities in the capsular polysaccharide structures, serotype 38 possesses distinct antigenic features that set it apart from serotypes 5 and 1. These unique structural elements are not recognized by the monoclonal antibodies, which could be due to differences in the oligosaccharide units or specific side groups attached to the polysaccharides.

The near-zero zeta potential of PnPS 38 suggests neutral charge or a zwitterion nature, which may potentiate a T cell-dependent B cell-mediated immune response. The interaction between ZPS-MHC class II and αßTCRs (T cell receptor) has been shown to drive the development of abscesses, demonstrating the integration of innate and adaptive immune responses.^47,50,52^ This characteristic might contribute to the observed cross-reactivity with polyclonal antibodies, as the immune system can generate a broader array of antibodies in response to a more complex antigenic structure.

These findings could enable the formulation of therapeutic strategies that are highly relevant for vaccine design. The cross-reactivity with polyclonal antibodies suggested that current vaccines targeting serotypes 5 and 1 might offer some degree of protection against serotype 38. However, the lack of reaction with monoclonal antibodies underscored the necessity for further research to identify the unique epitopes of serotype 38. The serotype 38 interaction with polyclonal and monoclonal antibodies from serotypes 5 and 1 enhanced our understanding of the immunological landscape of *S. pneumoniae*.

### 3.8 Critical Quality Attributes of Serotype 38 Capsular Polysaccharide

The determination of specific percentage limits for each functional group within polysaccharides is crucial to assess the integrity of immunogenic functional groups and ensure the preservation of a vaccine’s immunogenic properties. The biochemical characteristics of PnPS type 38 were evaluated by estimating the CQAs and proposing specific limits (Table 2). The key features and comparison of CPS repeating units and cross reactivity of Serotypes 38, 5, and 1 have been summarized in Table 3. The defined CQA limits provide a preliminary reference for assessing the purified capsular polysaccharide (CPS) quality of Type 38 produced by vaccine manufacturers. The assessment of critical quality attributes (CQAs) could be challenging with a single source of CPS. However, using ATCC capsular polysaccharide as a reference material is commonly employed as an internal standard for immunological assays by vaccine manufacturers; hence, the proposed tentative CQA limits could be validated further by comparing multiple sources of CPS of Spn type 38.

**Table 2:** Critical Quality Attribute Results of CPS of Spn Type 38

Quality attribute	Results	Proposing limits (concerning the ATCC CPS as a reference)
Molar mass (kg/mol)	768	≤800
Net charge (mV)	–0.7	Neutral (+10 to −10)
*O*-acetyl groups (% w/w)	5.8	≥6
Total nitrogen (%)	3.67	4 to 5
Total phosphorus (%)	0 (no peak)	0 to 1
Amino acid (serine) (relative % peak area by HPAEC-PAD)	7.8	≥8
Uronic acid (% w/w)	7.7	≥8
Hexosamines (relative % peak area by HPAEC-PAD)	9.78	≥10
Methyl pentose (%)	nil	nil

**Table 3:** CPS Structure, Antibody Reactivity, and Key Features of PnPs Type 38, 5, and Type 1

Features	PnPs 38	PnPs 1	PnPs 5
Repeating unit structure	→3)-[β-**D**-Gal*f*(1 → 2)]-β-**D**-Gal*p*A6(l-Ser)-(1 → 3)-α-**D**-Glc*p*NAc-(1 → 3)-α-**D**-Sug*p*-(1 → 4)-α-**D**-Gal*p*(2OAc)-(1 →	→3)-α-AATGalp-(1→4)-α-D-GalpA2_0.3_,3_0.3_Ac_2_-(1→3)-α-D-GalpA-(1→	[-4)-β-D-Glcp(1→4)-α-L-FucpNAc(1→3)-β-D-Sugp(1→]_n_ $\overset{3}{\underset{1}{↑}}$ α-L-PnepNAc(1→2)-β-D-GlcpA
Anti-PnPs1 reactivity	+	+	
Anti-PnPs5 reactivity	+		+
Keto sugar present	Sug_p_		Sug_p_
Rare sugar present		AATGalp	
Amino acid present	serine		
*O*-acetyls	yes (5.8% w/w)	yes	no
Net charge	–0.7 mV	–0.9 mV	–46.5 mV
Methyl pentose	no	no	no
Hexosamines present	yes		high
levorotatory hexosamines			yes

## 4 Conclusions

This study provides a comprehensive biochemical characterization of the newly prevalent nonvaccine serotype 38 of *S. pneumoniae*. We have characterized biochemical features, including size, molar mass, sugar composition, and O-acetyl content, and determined net charge of the serotype 38 in comparison to the net charge of PnPS 1 and nonzwitterionic PnPS 5. The immunological assessment, particularly the observed cross-reactivity with polyclonal antibodies from serotypes 5 and 1, underscores the potential for cross-protection. This highlights the significance of the inclusion of serotype 38 in vaccine development. Notably, the existence of an amino acid serine in the polysaccharide repeating unit of this serotype may play a vital role in host–pathogen interactions. The data presented in this study provide vital information for research and development of therapeutics, including vaccines. Together, addressing the challenge posed by nonvaccine serotypes like serotype 38 is vital for reducing the burden of IPD across all age groups. Further, the potential inclusion of serotype 38 in future vaccine formulations should be explored, and its spread along with impact on public health should be monitored.

## Data Availability

All data generated or analyzed during this study are included in this published article.

## Abbreviations

CPS: Capsular polysaccharide
CQA: Critical quality attributes
HPAEC-PAD/CD: High-performance anion-exchange chromatography with pulsed amperometry detection/conductivity detector
IPD: Invasive pneumococcal disease
DLS: Dynamic light scattering system
SEC-MALS-RI: Size exclusion chromatography connected with multiangle light scattering and refractive index detectors
Spn: *Streptococcus pneumonia*
PnPS: Pneumococcal polysaccharide
ZPS: Zwitterionic polysaccharide.
